# Catalysts of Enthusiasm in Teaching Older Adults how to use New Media: an Exploration of Pedagogical Approaches among Trainers of Older Adults in Poland

**DOI:** 10.1007/s10823-025-09549-6

**Published:** 2026-03-25

**Authors:** Łukasz Tomczyk, Izabela Kielar, Maria Lidia Mascia, Francisco David Guillen Gamez

**Affiliations:** 1 Jagiellonian University, Krakow, Poland; 2 University of Sassari, Sassari, Italy; 3 University of Malaga, Málaga, Spain

**Keywords:** Digital inclusion, Senior education, Digital competences, Teaching methods, Motivation, Geragogy

## Abstract

The article analyses effective methods of teaching older people how to use new technologies, with a particular emphasis on the role played by the attitudes and motivational skills of trainers. The aim of the research conducted in Poland in 2024 was to identify the methods, forms, and teaching aids considered by trainers to be the most effective in stimulating enthusiasm for learning. A qualitative analysis of 192 responses from participants in an e-learning course identified eleven categories of activities, among which the most frequently mentioned were learning by doing, an empathetic approach, educational games, group learning, and individual teaching. The results indicate that practical and problem-based methods, supported by multimedia tools (games, films), are the most effective in the process of increasing the digital inclusion of older people. The solutions used are in line with the constructivist, humanistic, and andragogical teaching paradigm, which emphasises personalisation, cooperation, and the active participation of learners. The conclusions from the research can form the basis for developing recommendations for institutions and trainers involved in the development of digital competences among older people.

## Introduction

The ageing of society and the progressive development of technology together pose a complex challenge to policy makers and public organization - particularly those in the educational sphere *(*Eurostat, 2024). On the one hand, there is a clear and pressing need to keep individuals in the labour market and to include older adults in the activities of the information society; on the other hand, we recognise the difficulties associated with effective digital education for adults - especially older adults, which, due to rapid and continuous development in the world of technology, tends to be oriented towards learning the technologies of yesterday. In addition, digital inclusion is significantly hampered by the internal barriers of older adults themselves (Tomczyk & Kielar, 2025). Research indicates that the main barriers to digital skills development among older adults are: a lack of need to use digital devices, counter-productive beliefs about the possibility of learning as an older adult, and fear of new technologies (Tomczyk et al., 2023a, b; Diana et al., 2025). Completing the picture are the results of studies showing older adults as vulnerable people who interpret learning difficulties as a sign of growing old faster than others; in such cases, older adults might show additional wariness around confronting this interpretation, as the fear of failure to learn about new technology might also translate into an increased fear of becoming older adults (Grotek & Kiliańska-Przybyło, 2012).

In this context, the effectiveness of the process of digital inclusion of older adults depends to a large extent on a number of non-measurable (or difficult to measure) issues that include the ability to motivate and provide support, willingness to express authentically positive feedback, and a kind of openness to see the value of the other person. This unique attitude that should be definitional among carers of older adults is based on the humanistic paradigm of didactics, derived from humanistic psychology, built on Rogers’ triad: empathy, acceptance, and congruence (Rogers, 1991), while not at all contradicting andragogical theory (Knowles, 1984), which postulates solution-oriented teaching for real problems.

In order to discover which teaching paradigms are preferred among future and current trainers of older adults, the subject of the study was a group of people interested in working on the digital inclusion of older adults and who had taken part in an e-learning course. The respondents answered an open-ended question related to the analysis of older adults’ needs and creative reflection in the context of generating enthusiasm and motivating action through teaching methods, forms, and means.

This article is an attempt to fill an empirical gap related to the professionalisation of geragogical activities aimed at digital inclusion.

## Overview of the Theoretical Framework

The education of older adults today represents one of the central issues of education as a profound pedagogical, psychological, and social opportunity that contributes to cognitive, emotional, and social well-being. A variety of theoretical frameworks help explain the mechanisms through which enthusiasm for learning can be cultivated among students, and some of these mechanisms are especially indicated to promote motivation and interest among older learners.

Intrinsic learner motivation is one of the pillars of educational success (Gottfried, 1985; Ryan & Deci, 2020; Taylor et al., 2014), while another is teacher attitude (often of even greater importance than political debates and education budgets (Hattie, 2009; Mahler et al., 2018). There are also studies on teacher enthusiasm influencing student performance (Frenzel et al., 2019). According to Self-Determination Theory (Deci & Ryan, 1985, 2002, 2017, 2020), intrinsic motivation is nurtured when learners experience autonomy, competence, and relatedness. Trainers who structure lessons to support these psychological needs—by offering meaningful tasks, celebrating progress, and encouraging collaborative learning—are more likely to ignite enthusiasm and sustain engagement. In the case of adult education, we are dealing with a situation in which the learner takes responsibility for their own learning process, and this means a heightened importance to their motivation, enthusiasm, and proactive attitude towards the teaching-learning process. Older adults, though, are characterised by a lack of self-confidence and motivation to learn new things. This makes the situation more complicated, which is why human relations coaching skills around effective motivation, appreciation, and encouragement are extremely important in the education of older adults.

It is evident that, in addition to the promotion of training and learning based on external factors of interest, it is imperative that older adults are made to feel enthusiastic about participation. This is influenced by a combination of personal and contextual factors, with the methodology employed and the relationship cultivated between teacher and student playing a fundamental role.

It is also important to promote learning to older adults to combat loneliness, to maintain their cognitive reserve, and to enhance their quality of life. Many studies underline the value of participation in a University of the Third Age (Schoultz et al., 2020; Opdebeeck et al., 2016). The U3A is an international movement for the education of people in late adulthood, which emerged from Peter Laslett’s concept of the ‘third age’. Within this approach, the third age is the period of life after retirement, which naturally lends itself to various types of leisure activities and lifelong learning (Laslett, 1987). U3As can function as diverse organisations focused on the local community (cultural centres, associations, religious associations, informal groups). The aim of such institutions is to run courses and interest groups, often without formal accreditation (Kobylarek et al., 2022). Such solutions are conducive to the co-creation of tailored educational content or the generation of course offerings by the participants who form a given organisation. When analysing the phenomenon of U3A or related organisations, attention should be paid to the numerous research findings that emphasise the contribution of these organisations to supporting the well-being, social capital, and agency of people in late adulthood (Swindell & Thompson, 1995). Furthermore, recent literature reviews highlight the importance of U3A in the practical implementation of local and global lifelong learning policies in the field of digital inclusion through the development of digital and media competences (Casanova et al., 2024; Gierszewski & Kluzowicz, 2021). These studies (Formosa, 2019; Casanova et al., 2024) can be an observatory of all those dynamics, both positive and negative, to be monitored, avoided, or promoted.

In all learning processes, but most important in the processes that apply to older adults, it is vital to consider the pedagogical approach to be adopted. Constructivist methodologies, which emphasise active learning and the co-construction of knowledge (Vygotsky, 1978; Bruner, 1996), have been shown to be particularly efficacious in adult education. These methods recognise learners as bearers of experience and encourage participation through dialogue, reflection, and problem-solving. In this regard, the andragogical model proposed by Malcolm Knowles (1984) emphasises that adult learners are internally motivated and self-directed, and that effective education should be experiential, relevant, and collaborative.

The body of research on learning later in life highlights the crucial role played by cognitive reserve and by cognitive and neurological processes, which tend to undergo progressive deterioration with advancing age. Among the most commonly reported difficulties are the reduction in resources for information processing, slower processing speed, decline in working memory capacity, and weakened attentional processes. These changes call for a rethinking of educational strategies to promote inclusion and learning success within the older population. Numerous studies emphasise how older adults tend to adopt compensatory strategies, often relying on familiar routines and cognitive patterns. This behaviour is consistent with the assumptions of Continuity Theory (Atchley, 1989), which posits that individuals tend to maintain behavioural, relational, and cognitive patterns developed earlier in life, even in old age, based on the belief that continuity fosters stability and subjective well-being. A further theoretical contribution comes from the SOC model (Selection, Optimization, and Compensation) developed by Baltes and Baltes (1990), which interprets aging as an adaptive process. According to this model, individuals select meaningful goals, optimise their remaining resources, and implement compensatory strategies to cope with cognitive and functional losses. This approach underscores the plasticity of human behaviour and the importance of creating flexible learning environments that enhance and support the capacities that remain active. Finally, Socioemotional Selectivity Theory (Carstensen, 1992) provides a perspective focused on motivation and emotion. According to this theory, as people age and become increasingly aware of the limited time ahead, they tend to focus their resources on emotionally meaningful activities and relationships. They prioritise positive experiences and invest in social contacts that promote well-being. This selectivity does not indicate social withdrawal; rather, it reflects a rational reorganisation of interpersonal interactions aimed at maximizing personal satisfaction and emotional stability.

All these theories and approaches offer an integrated interpretive framework that is highly valuable for designing educational interventions targeted at older adults. Promoting lifelong learning means not only supporting cognitive functioning, but also recognizing the emotional, motivational, and identity-related needs that are typical of this stage of life.

In the context of learning, it is fundamental to take into account all these peculiar aspects of what is generally termed the third age. In particular, the relational dimension between teacher and learner is critical to the process (Ljungblad, 2021). The body of research in the field of adult education (Merriam & Bierema, 2014) suggests that the cultivation of a positive, empathetic, and respectful relationship between instructors and learners has the potential to enhance the sense of belonging among learners and to reduce psychological barriers, such as fear of failure or feelings of inadequacy, which are often more pronounced in older adults returning to formal learning contexts (Villar et al., 2010). Trainers must be aware that older adults bring a whole life history with them to their lessons – something that is not generally the case with younger learners, and certainly not with the very young. Villar et al. (2010), in a study about teachers’ perspectives of older education, show that teachers reported an overwhelmingly positive experience, frequently highlighting a notable difference between the active, interested, and appreciative behaviour displayed by their older students and the relatively passive conduct demonstrated by their younger students. Furthermore, the teachers highlighted how they adapted their teaching methods to align with a more motivated and participative audience in classes designed for older individuals. This final consideration is also to be found in other similar and more recent studies (Tournier, 2022).

## Older adults, New media, and Pedagogical Approaches

The potential impact of technology on the aging process was a salient theme during the Covid-19 pandemic. However, many factors such as hearing impairment, cognitive impairments, and unfamiliarity with technology have the potential to compromise the efficacy of even the most sophisticated modern devices for older adults (Steinman et al., 2020; Tournier, 2022). Despite the rapid growth in the proportion of older adults using technology, access to new media still appears today to be limited and there are evidently gaps within populations. This digital divide, termed the ‘digital grey divide’, exhibits significant variations across different countries, attributable to a combination of individual, social, and cultural factors (Prensky, 2001; van Dijk, 2020; Niiranen, 2021).

The perceived usefulness of ICTs has been demonstrated to have an impact on their utilisation. A difference in the use of ICT before and after Covid-19 can be seen, with this due to the demonstration to older adults of the potential value of ICT functions and their importance (Tournier, 2022).

The Council of the European Union (2018) has updated the key competences for lifelong learning, including media literacy, which are fundamental in terms of an individual’s self-expression, health, employability, and social inclusion (Palsa & Salomaa, (2020). Media literacy is incorporated into educational curricula in order to facilitate learning, promote well-being, and enhance the quality of everyday life.

A recent systematic literature review conducted by Rasi et al. (2021) explores which aspects of media literacy are incorporated in practical interventions targeting older adults, the pedagogical methodologies employed to promote media skills among this demographic, and the practical educational implications that emerged. The results demonstrate that the majority of the interventions are aimed at enhancing the skills of older adults in using digital devices, technologies, and media for purposes such as information, health, learning, social communication, and entertainment. Dimensions related to understanding and producing media content appear to be addressed less frequently. The approaches employed appear to adapt to the specific needs of the participants (Vroman et al., 2015), with strong social support being a key factor in fostering learning.

In designing digital content instruction, designers may wish to consider interactions with new interfaces and applications. Emotional barriers such as technophobia, anxiety, or a perceived lack of utility can further discourage engagement. Fisk et al. (2020) articulate a set of foundational design principles tailored to the heterogeneous needs of aging populations. Central to their argument is advocacy for inclusive design that recognises the wide spectrum of age-related changes in cognitive, sensory, and motor functioning. The authors emphasise the necessity of simplicity in design, ensuring that interfaces are comprehensible and forgiving of user error. The authors underscore the importance of clarity of feedback and of a participatory design in order to enhance usability, satisfaction, and safety. Consequently, effective design must support psychosocial and pedagogical dimensions, facilitating independence and active participation.

However, the research shows that when provided with appropriate training and support, older adults can learn to use digital tools effectively, deriving significant benefits in terms of social connection, access to services, and cognitive stimulation (Czaja et al., 2019). This underscores the importance of inclusive pedagogical approaches tailored to the needs and contexts of aging learners.

## Methodology

### Aim and Subject of the Study

The aim of the study was to identify the perceptions of prospective and current Polish trainers of older adults about the different teaching methods, forms, and means they considered effective and that would generate enthusiasm for learning to use new technologies among the students in their charge. The respondents were participants in an e-learning course entitled: “How to effectively teach older people to use new technologies”, following which they responded anonymously to the following prompt as part of an open-ended question:

*Give three examples of effective teaching methods*,* forms*,* or tools that generate enthusiasm among older adults. Obviously*,* the activities listed should be related to the acquisition of digital competences.*

This instruction, firmly grounded in the knowledge acquired during the course, required participants to critically analyse the needs of older adults and reflect creatively on the generation of enthusiasm and the motivation of action through teaching methods, forms, and means. The study was a further step towards finding solutions to effectively neutralise internal barriers to digital inclusion for older adults (Bartol et al., 2021; Tomczyk et al., 2023a, b).

The following research questions were posed:

1. Which teaching methods do Polish trainers of older adults find most engaging, and why?
2. How do the teaching methods, forms, or tools preferred by trainers of older adults align with didactic paradigms intended to foster enthusiasm and engagement among learners?

### Research Procedure

#### Sample Description

**Fig. 1:**
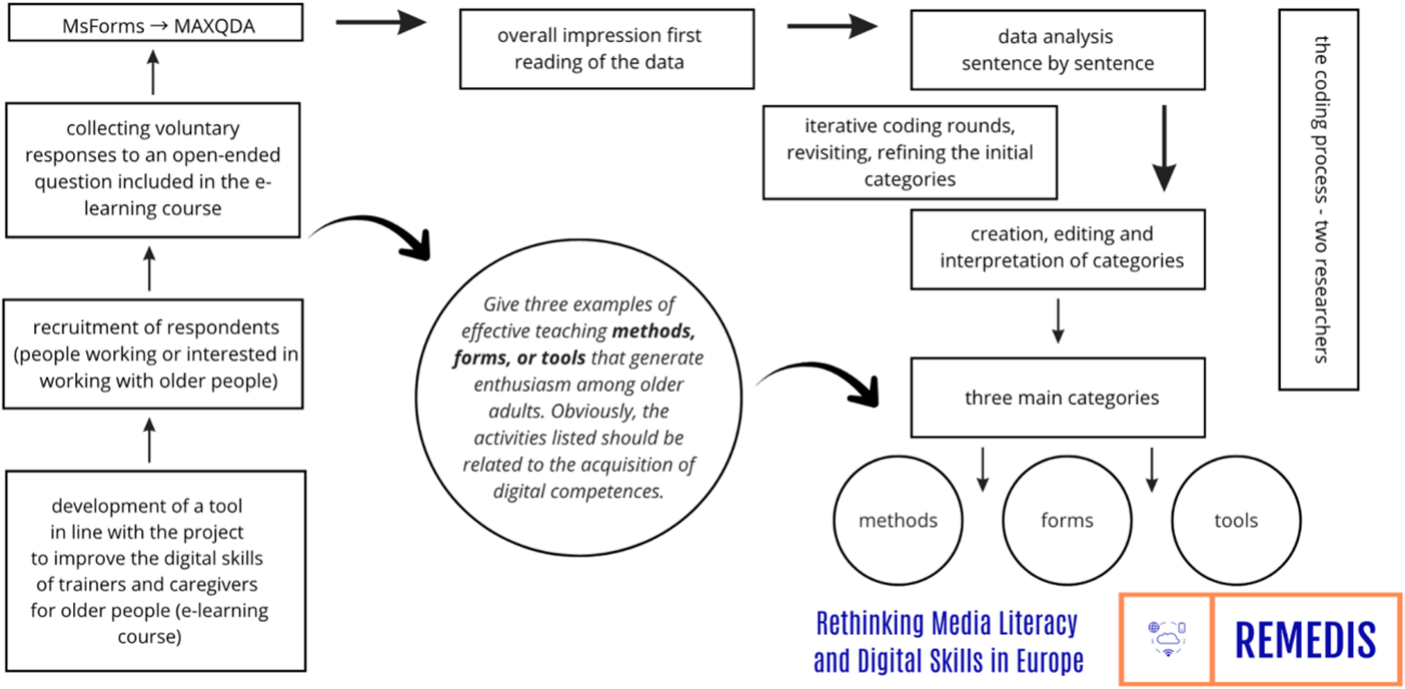
Procedure of qualitative data analysis

The coding covered a total of 192 responses - three responses each from 64 people aged 20 to 52 (7 men and 57 women). The respondents constituted a group of individuals interested in expanding their knowledge in the field of senior digital education. Some respondents had prior experience of working with older adults and were seeking to enhance their competences in teaching new technologies, while others possessed advanced digital skills but required further knowledge in the field of geragogy. In promoting the course, we primarily relied on the network of Universities of the Third Age as well as online platforms targeted at older adults. Consequently, the respondents were most likely to include trainers of older adults, caregivers, and individuals interested in fields such as andragogy and geragogy. Figure 1 shows the analysis process.

#### Data Analysis

The respondents answered the question which was delivered to them via Microsoft Forms, with their responses then being exported to MAXQDA Pro and analysed qualitatively. The question format allowed for preliminary selection of content, which facilitated the work of the coders. After analysis, the respondents’ statements were divided into three preliminary categories: methods, forms, and tools.

**Fig. 2:**
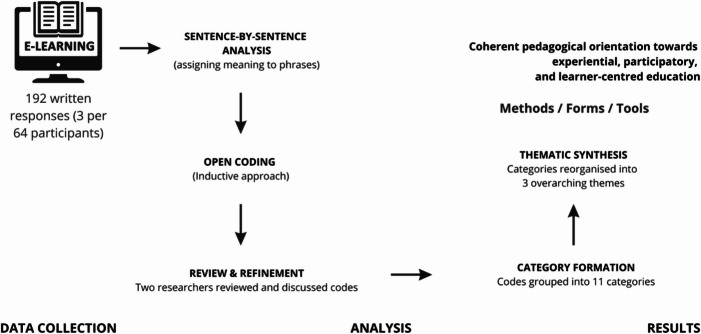
Diagram of the research procedure.

Given this preliminary selection, open coding was applied to facilitate a non-prescriptive analysis of each sentence, aimed at identifying as many recommendations as possible, including those extending beyond the knowledge acquired during the course. Figure 2 shows the research procedure.

This study received approval from the Ethics Committee of KU Leuven, which serves as the coordinating institution for the REMEDIS project. Ethical clearance was granted on 6 September 2023, under registration number G-2022-6106-R2(MAR).

The analysis was conducted sentence by sentence, assigning meanings to entire phrases, and the coding process was inductive (Konarzewski, 2000; Kubinowski, 2010; Gibbs, 2011). To ensure coding reliability, the coding process was reviewed by two researchers.

In the first stage of coding, 20 codes were identified and grouped into 11 categories, which were then analyzed and regrouped into three: methods, forms, and tools. Table 1 presents the coding system used to categorize the learning activities proposed by the respondents.

**Table 1:** Code system

No.	Codes	Code Count
1	Practical	62
2	Good atmosphere	30
3	Games	26
4	Collaboration	26
5	Benefits	23
6	Individual Teaching	14
7	Video	14
8	Experts	6
9	Lecture	5
10	Thematic Meetings	4
11	Mind maps	2
12	Staged performances	2
13	Brainstorm	2
15	Flashcards	1
16	Discussions	2
17	Reference to experience	1
18	Professionalism	1
19	Repeating content	1
20	Simple notes and pictures	1

## Results

Eleven categories were identified in this way.

*Learning By Doing* refers to direct practice, self-directed tasks, and problem solving. Example: [R1-1] “Organising hands-on workshops where older adults work on specific digital tasks or projects, with the support of mentors, can be very engaging. For example, workshops on creating and sharing photos online or learning how to use social media can be very rewarding and inspiring for older adults.”

*Empathy-Based Teaching* refers to the trainer’s distinctive approach towards the older adult, an approach full of empathy, patience, and building a positive atmosphere, which, according to the respondents, helps in overcoming barriers related to the fear of technology while also building self-confidence. Example [R1-52]: “Interactive workshops with an empathetic approach. Organising interactive workshops where older adults can learn in a friendly and supportive atmosphere. Using an empathetic approach from instructors who understand the concerns and challenges of learning new technologies at a later age. Creating a space where older adults feel accepted and understood, which can increase their motivation to learn.”

*Learning By Playing* refers to the use of games and free play on digital devices, which, according to the respondents, makes the learning process more attractive and engaging. Example [R3-43]: “Games and educational fun: The use of computer games, online quizzes or other interactive learning tools that teach basic digital skills in a fun and engaging way. Educational games can be a great learning tool because they allow the exploration of new topics in an interactive way, which can be particularly appealing to older adults.”

*Group Learning* refers to statements that mention collaborative (peer-to-peer) learning, as well as support groups fostering the sharing of experiences and building relationships, which significantly increases the comfort and engagement of older adults. Example [R3-27]: “Discussions are engaging and allow us to react immediately to opportunities that arise and to adapt methods to the specific group. But also, in the case of e.g. learning to use smartphones, it will allow older adults to compare how other phone models work. It allows them to see that, despite the differences, there are also a lot of similarities and that, should they need to change phone models, it is possible to transfer the skills learned in class to a new situation.”

*Purpose-Driven Learning* refers to mentions of the older adults’ personal life goals, the correct treatment of which should increase the meaningfulness of the training and the students’ motivation to learn. Example [R3-5]: “Allowing older adults to discover things that they themselves would like to learn from the internet. Whether it’s information from the world, hobby facts or just things to buy”.

*Individual Teaching* refers to statements about one-to-one teaching in which the pace and content of teaching can be fully tailored to the individual, which is particularly important when working with less advanced learners. Example [R1-40]: “Individual training sessions with mentors. Providing older adults with individual training sessions with experienced mentors can be a very effective motivational measure. Older adults can thus feel more comfortable and openly share their concerns and questions in a one-on-one meeting. Mentors can tailor the pace and content of the training to the individual, making the learning process more effective.”

*Video tutorial* refers to the use of video material. The respondents emphasised the usefulness of being able to play back the material more than once and thus for the learner to determine the speed at which they work through the material.

*Meetings With Experts* refers to the six statements that reported the value that older adults took from the opportunity to interact directly with people with expertise and that doing so would build trust and enhance motivation.

*Thematic Meetings* refers to focusing on specific issues (e.g. web security, banking applications) which facilitates learning through context and allows for practical application of the knowledge gained.

*Lecture* refers to the traditional form by which basic knowledge might be imparted. Lectures are more effective when they include interaction and are supported by examples from the learners’ daily lives, though it should be pointed out that adding interaction and personalization in this way suggests a move beyond what is traditionally considered a lecture.

Finally, *Other Solutions* refers to the proposition of a variety of individual solutions. This group includes printed materials, notes, role-play, case studies, and flashcards (also known as ‘fiches’), among others. Example [R2-60]: “Preparing so called ‘fiches’ to help remember the names of websites to be safe online. A fiche is a small piece of paper with an abbreviation on one side, e.g. *gov*, and on the other side an explanation that it is a *government* website”.

In the next step of the analysis, three main categories emerged: methods, forms, and didactic tools; the respondents’ suggestions were then assigned to the relevant category. The classification of didactic methods was adopted after the work of Wincenty Okoń (1995), who divides them into four groups: practical methods, problem-solving methods, methods of knowledge assimilation, and axiological methods. Analysis of respondents’ statements showed that Practical Methods were found to be the most effective in raising older adults’ engagement and enthusiasm (Table 2). 62 passages relating to this type of activity were encoded. Example: [R2-54] “Practical Exercises - Older adults can apply the knowledge they have gained to real life, e.g. find three things in a promotion on the *Biedronka* app that you could use for dinner tonight.” (*Biedronka* is one of the most popular supermarkets in Poland).

**Table 2:** Didactic methods proposed by respondents

No.	Didactic methods	Code Count
1	Practical Methods	62
2	Problem-Solving Methods	33
3	Knowledge Assimilation Methods	29
4	Axiological Methods	8

Problem-Solving Methods refer to such activities that help the learner to independently pursue knowledge and develop their skills, thus includign problem-solving, case study, and learning games.

Knowledge Assimilation Methods are mainly teacher activity-oriented and include lecture, presentation, and talk.

Axiological Methods are dominated by emotional activity through exposure to works of art or role-play. This group of methods was mentioned the least frequently.

In terms of forms of work, although many responses emphasised the individual approach and the need to work with a mentor or peer-to-peer (14 extracts were encoded in this way), many statements stressed the need to work in teams - sharing experiences and with mutual support (26 extracts were coded). Examples:

[R3-51] “Group activities and partnership programmes - organising regular group meetings where older adults can share their experiences, exchange knowledge, and solve problems related to the use of technology together.”

[R3-57] “Create support groups for older adults where they can share their experiences, help each other and benefit from the knowledge and skills of other participants.”

The following codes appeared in the teaching tools category (Table 3).

**Table 3:** Teaching tools proposed by respondents

No.	Teaching tools	Code Count
1.	Games	26
2.	Computer	24
3.	Film	21
4.	Smartphone	11
5.	Tablet	8

The analysis of the three main categories — methods, forms, and teaching tools — reveals a coherent pedagogical orientation towards experiential, participatory, and learner-centred education. The *methods* proposed by the respondents, dominated by practical and problem-solving approaches, highlight the importance of learning through doing, reflection, and contextual application. These methods prioritise active engagement and autonomy, allowing older adults to integrate new knowledge into their everyday routines.

The *forms* of work, in turn, emphasise the social and relational dimension of learning. Although individual mentoring was recognised as valuable, collaborative forms — particularly group learning and peer exchange — were viewed as key to sustaining motivation and overcoming technological anxiety. The respondents thus stressed that emotional safety and mutual support were as crucial as the transmission of knowledge itself.

Finally, the *tools* mentioned by the respondents support these pedagogical choices: interactive and multimedia resources such as games, films, and smartphones not only facilitate self-paced learning but also stimulate curiosity and enjoyment. Together, these three dimensions suggest that effective digital education for older adults integrates practical, relational, and technological components, forming a holistic model that aligns with constructivist and humanistic assumptions about adult learning.

As a result of the analysis and interpretation of the data collected, the research questions were answered.

1. Which teaching methods do trainers of older adults find most engaging, and why?

Respondents found Practical Methods and Problem-Solving Methods (characterised by learner-centred approaches) to be far more likely to generate enthusiasm among older adults.

2. How do the teaching methods, formats, or tools preferred by trainers of older adults align with didactic paradigms intended to foster enthusiasm and engagement among learners?

The methods chosen by the respondents are in line with Knowles’ theory and the humanistic paradigm, as well as constructivist didactics.

## Discussion

Digital training for adults, and especially for older adults, is essential for their full inclusion in the information society and their continued active participation in the labour market. However, this training process is not without its challenges, given the diverse backgrounds, motivations, and technological barriers these individuals face. In this context, it is essential to incorporate the perspective of those who act as trainers in these processes, as they possess a grounded understanding of the needs, interests, and learning styles of adult learners. Their experience allows us to identify the most effective pedagogical approaches and adapt teaching paradigms to foster interest, participation, and the meaningful acquisition of digital skills. Recognising their role not only in the implementation but also in the design of training strategies is key to moving toward more inclusive and sustainable models of digital education for adults. With that in mind, the purpose of this study was to investigate which teaching methods or forms generate enthusiasm among educators of older adults, and in which didactic paradigm these training strategies fit, according to experience of such educators.

### Motivational Methods

Based on the qualitative analysis of the responses to the survey, it has been found that the *Learning by Doing* approach is the method most valued by trainers for teaching older adults, along with the support of mentors. Furthermore, it is evident that practical learning has a positive impact in terms of the learning outcomes of students who learn about digital software (Sitompul, 2021), as well as in the development of creativity and useful ideas, with improvements found in both problem-solving skills and communication skills (Desrani et al., 2024).

Another effective method found by the participants was *Empathy-Based Teaching*, an approach focused on empathy, patience, and creating a positive, caring environment. Trainers cite this teaching style as a successful educational approach, as it helps their learners overcome their initial fear of technology and strengthen their confidence in learning. The literature shows how teachers who practice authentic care, respond to socio-emotional needs, and seek to connect with their students achieve greater motivation, a sense of belonging, and active participation on the part of their students (Tang et al., 2024), even managing to rebuild meaningful relationships in the remotest environments (Miller, 2021). All of which suggests the usefulness of more flexible, human, and accessible learning (Sangiuliano et al., 2023).

Two other motivational strategies the trainers highlighted were *Learning by Playing*, which uses games to make digital learning more engaging and participatory, as well as *Group Learning*, which utilises collaborative learning approaches. This not only increases the engagement of older students but also promotes the development of key skills. As Pasieka et al. (2023) point out, game-based activities contribute to improving motor skills, attention, logic, spatial thinking, and creativity, while promoting perseverance and the ability to work both independently and in teams. Furthermore, recent studies show that learning by solving puzzles and participating in educational games has a significant impact on student performance in technological areas, such as cybersecurity (Khan et al., 2022), which underlines the potential of these methods in the digital training of older adults.

Another relevant approach that emerges from the results is the so-called *Purpose-Driven Learning*. In the context of digital education, this method allows older adults not only to acquire technical skills, but also to clearly visualise how these skills can improve their daily lives, whether by accessing information about their hobbies, facilitating communication with family members, or carrying out online tasks independently. This focus on personal benefits reinforces intrinsic motivation, a crucial aspect of older adult education, where learning is most effective when linked to practical and meaningful goals (Ryan & Deci, 2020). Furthermore, by allowing the students themselves to define their learning objectives, Purpose-Driven Learning aligns with Knowles’ (1984) andragogical principles, promoting self-directed learning oriented toward solving real problems.

Another aspect worth highlighting is the importance of *Individual Teaching* in the digital learning processes of older adults. This personalised teaching method allows the pace, content, and strategies to be adapted to the specific needs and abilities of each participant (Varier et al., 2017), thus overcoming the barriers that more standardized methods often present. In this context, video tutorials emerge as a particularly useful resource to enhance individual learning (Tomczyk et al., 2023a, b). As Dahlan et al. (2023) states, video tutorials, by offering clear, repeatable, and visually accessible explanations, can allow each person to advance autonomously, reviewing the content as many times as required and at the time that is most convenient for them. Furthermore, by combining this method with in-person sessions or personalized tutoring, they can strengthen the blended approach of Individual Teaching, providing a scaffolding that facilitates progression from initial dependency to more self-directed learning. In this way, individualized teaching, supported by digital resources such as video tutorials, not only optimises skill acquisition but also respects and values ​​the unique learning experiences and styles of each older adult.

Finally, the results also highlight the value of *Meetings With Experts* and *Thematic Meetings* as complementary strategies that enrich the digital learning process for older adults. Both methods share the virtue of creating spaces where participants can delve into specific topics and engage directly with professionals. These thematic meetings promote not only the transmission of technical information but also the exchange of experiences, which contributes to strengthening confidence and a sense of belonging among older adults (Calcaterra et al., 2022).

### Didactic Methods

The first method most valued by trainers for teaching older adults was the use of *Practical Methods*, considered the most effective in generating enthusiasm and commitment. This result aligns with other studies that have highlighted the value of practical activities in adult education. Raximovna (2022) highlights that this approach allows students to connect the theoretical information presented in lessons with daily life, while also employing a variety of strategies to strengthen useful skills at personal and professional levels. Along the same lines, Tiberghien (2000) describes practical work as a means of linking two essential domains of knowledge: the observable world and the world of ideas. However, Abrahams and Millar (2008) caution that the effectiveness of practical work depends on achieving this balance, as they found significant differences when only the observable was addressed without connection to the conceptual plane. In the case of digital training for older adults, practical activities, such as the specific exercise of searching for products in mobile applications, not only facilitate the transfer of learning to real-life situations, but also increase the confidence of participants, as the authors Whittle and Bickerdike (2015) point out in the findings of their study. In this way, practical methods emerge not only as effective pedagogical strategies, but also as key drivers of motivation and empowerment in the training of older adults.

The second most highly valued method by trainers for teaching older adults was the use of *Problem-Solving Methods*, which encompasses activities such as problem-solving, case studies, and learning through games. These methods allow adult learners to autonomously seek new knowledge and develop practical skills, as exemplified in the dynamics of competitive games where participants evaluate the authenticity of online information. The effectiveness of this approach is widely supported in the literature. Simanjuntak et al. (2021) demonstrate that problem-based learning, especially when combined with simulations, has a positive impact on the simultaneous development of problem-solving and creative thinking skills, competences that are increasingly necessary in a digitalized context. Indeed, Lee et al. (2017) highlight that problem-based learning (PBL) is configured as an effective method that encourages active learning through authentic problem solving. Wanya (2016) adds that a person with good problem-solving skills not only applies correct solutions, but is also able to evaluate the validity and reasonableness of their responses, a crucial ability when it comes to discerning the reliability of information in digital environments. Furthermore, Unal and Cakir (2021) find that collaborative problem-solving, supported by Web 2.0 technologies, significantly improves both learners’ performance and engagement. Thus, Problem-Solving Methods emerge as a key way to enhance not only autonomous learning in older adults, but also to strengthen their critical thinking and active participation in digital training activities.

The third teaching strategy to be highlighted was *Knowledge Assimilation Methods*, which include activities focused primarily on the teacher’s actions, such as lectures, presentations, and introductory talks. These methods allow the trainer to structure the transmission of knowledge and contextualise the information they present, as reflected in sessions that combine a brief professional talk with motivating anecdotes that foster a friendly and stimulating atmosphere. The literature confirms that when these methods are well designed, they can maintain high levels of attention and engagement. (Khalil et al., 2018) point out that lectures, traditionally seen as passive, can be transformed into powerful learning instruments if interactive elements, narratives, and visual resources are incorporated that capture attention and facilitate the assimilation of knowledge.

Finally, *Axiological Methods*, although less frequently mentioned by the respondents, play an important role in mobilising the emotional dimension of learning, mainly through exposure to artistic works or audiovisual performances. These strategies seek to foster empathy and strengthen older adults’ identification with shared experiences, as is the case with showing films that portray other older adults’ digital learning journeys. Recent research supports this perspective. Niemi and Multisilta (2016) highlight that methods such as digital storytelling not only promote critical reflection but also enhance empathy and the construction of personal meaning, essential aspects in intergenerational learning contexts. Along these lines, Robin (2016) emphasizes that digital storytelling allows learners to connect emotionally with the content, thus promoting greater retention and engagement. In this way, axiological methods emerge as a complementary approach that enriches learning in older adults by integrating the emotional and ethical dimensions with the development of digital competences.

Previous research in Western Europe has often linked older adults’ participation in educational programs mainly to socialisation and community engagement. For instance, Formosa (2019) and Formosa (2014) described the Universities of the Third Age as spaces that promote active and productive aging through empowerment and autonomy. Similarly, Villar et al. (2010) have found that Spanish teachers value emotional connection as a supportive element in their classes. However, in our study, Polish trainers placed much stronger emphasis on emotional support and empathy as the main motivational factors. This suggests that the affective dimension of teaching may play a more central role in contexts where interpersonal care and solidarity are important cultural values. Therefore, this study adds to previous research by highlighting the emotional and relational side of digital training for older adults, a factor that has not been widely discussed before in geragogical studies. This approach offers a more human view that can help shape digital education policies to be more inclusive and sensitive to the needs of older learners.

## Conclusions

Digital competence training for older adults is key to bridging the intergenerational digital divide and promoting full social inclusion in the information society (Tomczyk & Kielar, 2025). To achieve this, equitable access to technologies should not be limited to the younger generations but should also extend to those who, for structural or cultural reasons, have been left behind (Gitto, 2021).

The results of this study reveal that the most effective teaching methods for motivating older adults in digital learning are Learning by Doing, Empathy-Based Teaching, Learning by Playing, and Group Learning. These methods are embodied in practical teaching methods that focus on action, empathy, and collaboration, which trainers consider the most conducive to generating enthusiasm and engagement (Tomczyk et al., 2023a, b). In particular, the importance of creating safe, caring, and personalised environments reinforces the need to apply a humanistic approach based on Rogers’ (1991) triad: empathy, acceptance, and authenticity. Furthermore, group work and recreational activities help reduce fear of technology and promote collaborative learning (Villar et al., 2010; Pasieka et al., 2023). This evidence confirms that digital learning among older adults is enhanced by experiential, participatory, and emotionally meaningful strategies. Likewise, the results show that the trainer’s enthusiasm has a direct impact on older adults’ attitudes and motivation toward digital learning, supporting what Hattie (2009) and Frenzel et al. (2019) have reported. Trainers who structure their classes based on the psychological needs of autonomy, competence, and connection are able to sustain their students’ intrinsic motivation (Deci & Ryan, 2002). This attitude is especially relevant for overcoming the emotional barriers that many older adults face when dealing with technology.

The study’s focus on a single geographic context (Poland) and a relatively homogeneous sample appear to be a limitation that stands in the way of any generalisation of the results. Future work should expand the research to other countries and cultural profiles to explore how variables such as gender, rural-urban environment, or educational level affect digital motivation in older adults. Furthermore, it would be useful to conduct longitudinal research to determine whether the initial enthusiasm fostered by active methods is sustained over time. The perceptions of older adults themselves could also be explored, to contrast with the views of their trainers. All of this would open up new avenues for consolidating more personalised and sustainable training proposals.

From a theoretical and practical perspective, the results of this study reinforce the validity of paradigms such as Knowles’ (1984) and Rogers’ (1991) humanistic model, applied to digital teaching with older adults. In practice, this translates into the need to design strategies focused on empathy, personalised learning, and the use of methods such as “learning by doing,” which have proven to be highly motivating. The implication here is that teachers of older adults should be given training about the specific emotional and pedagogical competences for gerontagogical work. Furthermore, this evidence can guide more effective public policies regarding intergenerational digital inclusion.

In addition to the pedagogical implications arising from the nature of the study’s objectives, the analyses presented provide several important and practical insights for curriculum developers, policy makers, and institutions involved in European digital inclusion initiatives (Abad, 2014). The results suggest that training modules for older people should include structured mentoring systems, collaborative workshops, and educational tasks using solutions that meet the real needs of this group. This praxeological approach is consistent with senior policies based on solutions close to real-life situations and the creation of a friendly learning environment (Russo, 2025; Kużelewska et al., 2025). In addition, Universities of the Third Age, non-governmental organisations, senior clubs, and local cultural centres could use the results presented to adapt curricula that build the confidence and autonomy of older people, support the resolution of everyday challenges, and provide an opportunity to improve their quality of life. The strategies presented in the article are consistent with the latest research that emphasises the importance of an empathy based approach and the co-creation of digital inclusion solutions (Paluch et al., 2023; Pihlainen et al., 2022).

Also attention should be paid to the methodological limitations resulting from the approach adopted in data collection. The research sample was limited to one country, i.e. participants from Poland. This is a significant limitation that prevents the generalisation of the presented results to different cultural and socio-economic contexts. Furthermore, although the qualitative nature of the data allows for an in-depth analysis of micro-perspectives, it does not provide quantitative confirmation of the observed trends (by assessing the effectiveness of individual solutions using, for example, effect size). Taking into account the conclusions and recommendations, future research should therefore include comparative international analyses with additional consideration of mixed-method approaches (including pre-test and post-test). Such solutions will significantly strengthen the empirical basis for accurate and confirmed recommendations on methodologies for working with older adults in the field of digital inclusion. In addition, longitudinal projects are also useful in determining whether enthusiasm and motivation are sustained over time when using the aforementioned methodological solutions, and whether the identified teaching methods actually sustain the engagement of seniors in the digital world (Miller et al., 2024).

## Data Availability

No datasets were generated or analysed during the current study.

## References

[CR1] Abad, L. (2014). Media literacy for older people facing the digital divide: The e-inclusion programmes design. *Comunicar*, *21*(42), 173–180. 10.3916/c42-2014-17

[CR2] Abrahams, I., & Millar, R. (2008). Does practical work really work? A study of the effectiveness of practical work as a teaching and learning method in school science. *International Journal of Science Education*, *30*(14), 1945–1969. 10.1080/09500690701749305

[CR3] Atchley, R. C. (1989). A continuity theory of normal aging. *The Gerontologist*, *29*(2), 183–190. 10.1093/geront/29.2.183 2519525 10.1093/geront/29.2.183

[CR4] Baltes, P. B., & Baltes, M. M. (1990). Psychological perspectives on successful aging: The model of selective optimization with compensation. In P. B. Baltes & M. M. Baltes (Eds.). *Successful aging: Perspectives from the behavioral sciences* (pp. 1–34). Cambridge University Press. 10.1017/CBO9780511665684.003

[CR5] Bartol, A., Herbst, J., & Pierścińska, A. (2021). *Socio-digital exclusion in Poland. Status of the phenomenon, trends, recommendations* [Report]. Orange Foundation. https://fundacja.orange.pl/app/uploads/2021/11/RAPORT_WYKLUCZENIE-SPOLECZNO-CYFROWE-W-POLSCE_2021.pdf

[CR73] Bruner, J. (1996). *The culture of education*. Cambridge, MA: Harvard University Press.

[CR6] Calcaterra, V., Panciroli, C., & Sala, M. (2022). Group and community work in practice: Students learning from experts-by-experience. *Italian Journal of Sociology of Education*, *14*(1), 113–131. 10.14658/PUPJ-IJSE-2022-1-7

[CR7] Carstensen, L. L. (1992). Social and emotional patterns in adulthood: Support for socioemotional selectivity theory. *Psychology and Aging,**7*(3), 331–338. 10.1037/0882-7974.7.3.331 1388852 10.1037//0882-7974.7.3.331

[CR8] Casanova, G., Weil, J., & Cerqueira, M. (2024). The evolution of universities of the third age around the world: A historical review. *Gerontology & Geriatrics Education*, *45*(3), 483–498. 10.1080/02701960.2023.2231375 37408316 10.1080/02701960.2023.2231375

[CR75] Council of the European Union. (2018). Council recommendation of 22 May 2018 on key competences for lifelong learning (2018/C 189/01). *Official Journal of the European Union C, 189*, 1–13.

[CR9] Czaja, S. J., Boot, W. R., Charness, N., & Rogers, W. A. (2019). *Designing for older adults: Principles and creative human factors approaches* (3rd ed.). CRC. 10.1201/b22189

[CR10] Dahlan, M. M., Halim, N. S. A., Kamarudin, N. S., & Ahmad, F. S. Z. (2023). Exploring interactive video learning: Techniques, applications, and pedagogical insights. *International Journal of Advanced and Applied Sciences,**10*(12), 220–230. 10.21833/ijaas.2023.12.024

[CR69] Deci, E. L., & Ryan, R. M. (1985). *Intrinsic motivation and self-determination in human behavior*. New York, NY: Plenum.

[CR70] Deci, E. L., & Ryan, R. M. (2002). *Handbook of self-determination research. Rochester*, NY: University of Rochester Press.

[CR11] Desrani, A., Ritonga, A. W., & Lubis, M. (2024). Learning by doing: A teaching paradigm for active learning in Islamic high school. *Journal of Education and e-Learning Research,**10*(4), 793–799. 10.20448/jeelr.v10i4.5224

[CR12] Diana, M. G., Mascia, M. L., Tomczyk, Ł, & Penna, M. P. (2025). The digital divide and the older adults: How urban and rural realities shape well-being and social inclusion in the Sardinian context. *Sustainability,**17*(4), Article 1718. 10.3390/su17041718

[CR13] Eurostat (2024). *Ageing Europe-Statistics on population developments*. Retrieved from https://ec.europa.eu/eurostat/statistics-explained/index.php?title=Ageing_Europe_-_statistics_on_population_developments

[CR14] Fisk, A. D., Czaja, S. J., Rogers, W. A., Charness, N., & Sharit, J. (2020). *Designing for older adults: Principles and creative human factors approaches*. CRC.

[CR15] Formosa, M. (2014). Four decades of universities of the third age: Past, present, future. *Ageing and Society,**34*(1), 42–66. 10.1017/S0144686X12000797

[CR16] Formosa, M. (Ed.). (2019). *The university of the third age and active ageing: European and Asian-Pacific perspectives*. Springer. 10.1007/978-3-030-21515-6

[CR17] Frenzel, A. C., Taxer, J. L., Schwab, C., & Kuhbandner, C. (2019). Independent and joint effects of teacher enthusiasm and motivation on student motivation and experiences: A field experiment. *Motivation and Emotion,**43*(2), 255–265. 10.1007/s11031-018-9738-7

[CR18] Gibbs, G. (2011). *Analizowanie Danych jakościowych*. Wydanie pierwsze. Wydawnictwo Naukowe PWN.

[CR19] Gierszewski, D., & Kluzowicz, J. (2021). The role of the university of the third age in meeting the needs of older adult learners in Poland. *Gerontology & Geriatrics Education*, *42*(3), 437–451. 10.1080/02701960.2021.1871904 33423601 10.1080/02701960.2021.1871904

[CR20] Gitto, L. (2021). Ability and frequency of ICTs use in an older adults’ sample: Implications for developing an active aging educational strategy. *International Journal of Knowledge and Learning*, *14*(1), 86–100. 10.1504/IJKL.2021.115035

[CR21] Gottfried, A. E. (1985). Academic intrinsic motivation in elementary and junior high school students. *Journal of Educational Psychology,**77*(6), 631–645. 10.1037/0022-0663.77.6.631

[CR22] Grotek, M., & Kiliańska-Przybyło, G. (2012). The role of affective factors in the process of learning a foreign language by senior citizens. *Teraźniejszość - Człowiek - Edukacja*,*3*. https://opus.us.edu.pl/info/article/USL191cc79b334f461db02636507b3d6771/.

[CR23] Hattie, J. (2009). *Visible learning: A synthesis of over 800 meta-analyses relating to ACHIEVEMENT*. Routledge.

[CR24] Khalil, M. K., Abdel Meguid, E. M., & Elkhider, I. A. (2018). Teaching of anatomical sciences: A blended learning approach. *Clinical Anatomy,**31*(3), 323–329. 10.1002/ca.23052 29352730 10.1002/ca.23052

[CR25] Khan, M. A., Merabet, A., Alkaabi, S., & Sayed, H. E. (2022). Game-based learning platform to enhance cybersecurity education. *Education and Information Technologies,**27*(4), 5153–5177. 10.1007/s10639-021-10807-6

[CR26] Knowles, M. (1984). *Andragogy in Action. Applying modern principles of adult education*. Jossey Bass.

[CR27] Kobylarek, A., Błaszczyński, K., Ślósarz, L., Madej, M., Carmo, A., Hlad, Ľ, & Petrikovičová, L. (2022). The quality of life among university of the third age students in Poland, Ukraine and Belarus. *Sustainability,**14*(4), Article 2049. 10.3390/su14042049

[CR28] Konarzewski, K. (2000). *How to do educational research: A practical methodology*. Wydawnictwa Szkolne i Pedagogiczne.

[CR29] Kubinowski, D. (2010). *Qualitative pedagogical research: Philosophy, methodology, evaluation*. Wydawnictwo Uniwersytetu Marii Curie-Skłodowskiej.

[CR30] Kużelewska, E., Tomaszuk, M., & Malinowski, D. (2025). The elderly digital divide: digital exclusion versus the right not to use the internet. *International Journal for the Semiotics of Law-Revue Internationale De Sémiotique Juridique*, 1–20.

[CR31] Laslett, P. (1987). The emergence of the third age. *Ageing and Society*, *7*(2), 133–160. 10.1017/s0144686x00012538

[CR32] Lee, M. N., Nam, K. D., & Kim, H. Y. (2017). Effects of simulation with problem-based learning program on metacognition, team efficacy, and learning attitude in nursing students: Nursing care with increased intracranial pressure patient. *CIN: Computers, Informatics, Nursing,**35*(3), 145–151. 10.1097/CIN.0000000000000308 27820714

[CR33] Ljungblad, A. L. (2021). Pedagogical relational teachership (PeRT)–a multi-relational perspective. *International Journal of Inclusive Education,**25*(7), 860–876. 10.1080/13603116.2019.1581280

[CR34] Mahler, D., Großschedl, J., & Harms, U. (2018). Does motivation matter? - The relationship between teachers’ self-efficacy and enthusiasm and students’ performance. *PLoS ONE*. 10.1371/journal.pone.0207252 10.1371/journal.pone.0207252 PMC6248951 30462713

[CR74] Merriam, S. B., & Bierema, L. L. (2014). *Adult learning: Linking theory and practice *(1st ed.). San Francisco, CA: Jossey-Bass, a Wiley brand.

[CR35] Miller, K. E. (2021). A light in students’ lives: K-12 teachers’ experiences (re) building caring relationships during remote learning. *Online Learning,**25*(1), 115–134. 10.24059/olj.v25i1.2486

[CR36] Miller, L. M. S., Callegari, R. A., Abah, T., & Fann, H. (2024). Digital literacy training for low-income older adults through undergraduate community-engaged learning: Single-group pretest-posttest study. *JMIR Aging,**7*, Article e51675. 10.2196/51675 38599620 10.2196/51675 PMC11134247

[CR37] Niemi, H., & Multisilta, J. (2016). Digital storytelling promoting twenty-first century skills and student engagement. *Technology Pedagogy and Education*, *25*(4), 451–468. 10.1080/1475939X.2015.1074610

[CR38] Niiranen, S. (2021). Supporting the development of students’ technological understanding in craft and technology education via the learning-by-doing approach. *International Journal of Technology and Design Education,**31*(1), 81–93. 10.1007/s10798-019-09546-0

[CR77] Okoń, W. (1995). *Wprowadzenie do dydaktyki ogólnej (Introduction to General Didactics)*. Warszawa: Wydawnictwo Akademickie Żak.

[CR39] Opdebeeck, C., Martyr, A., & Clare, L. (2016). Cognitive reserve and cognitive function in healthy older people: A meta-analysis. *Aging, Neuropsychology, and Cognition,**23*(1), 40–60. 10.1080/13825585.2015.1041450 10.1080/13825585.2015.1041450 25929288

[CR40] Palsa, L., & Salomaa, S. (2020). Media literacy as a cross-sectoral phenomenon: Media education in Finnish ministerial-level policies. *Central European Journal of Communication*, *13*(2), 162–182. 10.19195/1899-5101.13.2(26).2

[CR41] Paluch, R., Cerna, K., Kirschsieper, D., & Müller, C. (2023). Practices of care in participatory design with older adults during the COVID-19 pandemic: Digitally mediated study. *Journal of Medical Internet Research,**25*, Article e45750. 10.2196/45750 37459177 10.2196/45750 PMC10390970

[CR42] Pasieka, N., Romanyshyn, Y., Chupakhina, S., Matveieva, N., Zakharasevych, N., & Pasieka, M. (2023). Lego technology as a means of enhancing the learning activities of junior high school students in the conditions of the New Ukrainian School. In *Lecture Notes in Networks and Systems* (Vol. 633, pp. 530–541). Springer. 10.1007/978-3-031-26876-2_51

[CR43] Pihlainen, K., Ehlers, A., Rohner, R., Cerna, K., Kärnä, E., Hess, M., Hengl, L., Aavikko, L., Frewer-Graumann, S., Gallistl, V., & Müller, C. (2022). Older adults’ reasons to participate in digital skills learning: An interdisciplinary, multiple case study from Austria, Finland, and Germany. *Studies in the Education of Adults*, *55*(1), 101–119. 10.1080/02660830.2022.2133268

[CR44] Prensky, M. (2001). Digital natives, digital immigrants part II: Do they really think differently? *On the Horizon,**9*(6), 1–6. 10.1108/10748120110424843

[CR78] Rasi, P., Vuojäärvi, H., & Rivinen, S. (2021). Promoting media literacy among older people: A systematic review. *Adult Education Quarterly, 71*(1), 37–54. 10.1177/0741713620923755

[CR45] Raximovna, T. F. (2022). Didactic and motivational opportunities for the use of variable approaches to increase the professional competence of future defectologists. *ACADEMICIA: An International Multidisciplinary Research Journal,**12*(4), 738–740. 10.5958/2249-7137.2022.00352.4

[CR46] Robin, B. R. (2016). The power of digital storytelling to support teaching and learning. *Digital Education Review,**30*, 17–29. 10.1344/der.2016.30.17-29

[CR47] Rogers, C. (1991). *Client-centred therapy. Meeting groups*. Thesaurus-.

[CR48] Russo, M. (2025). Identifying digital active ageing policies in the EU: The case of Italy. *E-Journal of International and Comparative Labour Studies*, *14*(1).

[CR71] Ryan, R. M., & Deci, E. L. (2017). *Self-determination theory: Basic psychological needs in motivation, development, and wellness*. New York, NY: Guilford Press.

[CR49] Ryan, R. M., & Deci, E. L. (2020). Intrinsic and extrinsic motivation from a self-determination theory perspective: Definitions, theory, practices, and future directions. *Contemporary Educational Psychology,**61*, Article 101860. 10.1016/j.cedpsych.2020.101860

[CR50] Sangiuliano Intra, F., Nasti, C., Massaro, R., Perretta, A. J., Di Girolamo, A., Brighi, A., & Biroli, P. (2023). Flexible learning environments for a sustainable lifelong learning process for teachers in the school context. *Sustainability,**15*(14), Article 11237. 10.3390/su151411237

[CR51] Schoultz, M., Öhman, J., & Quennerstedt, M. (2020). A review of research on the relationship between learning and health for older adults. *International Journal of Lifelong Education*, *39*(5–6), 528–544. 10.1080/02601370.2020.1819905

[CR52] Simanjuntak, M. P., Hutahaean, J., Marpaung, N., & Ramadhani, D. (2021). Effectiveness of Problem-Based Learning combined with computer simulation on students’ problem-solving and creative thinking skills. *International Journal of Instruction,**14*(3), 519–534. 10.29333/iji.2021.14330a

[CR53] Sitompul, H. (2021). Development of media using Adobe flash CS6 with a learning by doing approach sewing technology in the student grade X vocational high school of fashion design. *6th annual international seminar on transformative education and educational leadership (AISTEEL 2021)* (pp. 608–618). Atlantis.

[CR54] Steinman, M. A., Perry, L., & Perissinotto, C. M. (2020). Meeting the care needs of older adults isolated at home during the COVID-19 pandemic. *JAMA Internal Medicine,**180*(6), 819–820. 10.1001/jamainternmed.2020.1661 32297903 10.1001/jamainternmed.2020.1661

[CR55] Swindell, R., & Thompson, J. (1995). An international perspective on the university of the third age. *Educational Gerontology*, *21*(5), 429–447. 10.1080/0360127950210505

[CR56] Tang, A. L., Walker-Gleaves, C., & Rattray, J. (2024). University students’ conceptions and experiences of teacher care amidst online learning. *Teaching in Higher Education,**29*(2), 366–387. 10.1080/13562517.2021.1989579

[CR57] Taylor, G., Jungert, T., Mageau, G. A., Schattke, K., Dedic, H., Rosenfield, S., & Koestner, R. (2014). A self-determination theory approach to predicting school achievement over time: The unique role of intrinsic motivation. *Contemporary Educational Psychology,**39*(4), 342–358. 10.1016/j.cedpsych.2014.08.002

[CR58] Tiberghien, A. (2000). Designing teaching situations in the secondary school. In R. Millar, J. Leach, & J. Osborne (Eds.), *Improving science education: The contribution of research* (pp. 27–47). Open University.

[CR59] Tomczyk, Ł., & Kielar, I. (2025). Neutralising external and internal barriers in the digital inclusion process for Seniors-Finding ways to effectively shape digital and media competences among older people. *Technology Knowledge and Learning*, 1–18. 10.1007/s10758-024-09813-7

[CR61] Tomczyk, Ł, Mascia, M. L., Gierszewski, D., & Walker, C. (2023b). Barriers to digital inclusion among older people: A intergenerational reflection on the need to develop digital competences for the group with the highest level of digital exclusion. *Innoeduca. International Journal of Technology and Educational Innovation,**9*(1), 5–26. 10.24310/innoeduca.2023.v9i1.16433

[CR60] Tomczyk, Ł, Mascia, M. L., & Guillen-Gamez, F. D. (2023a). Video tutorials in teacher education: Benefits, difficulties, and key knowledge and skills. *Education Sciences,**13*(9), Article 951. 10.3390/educsci13090951

[CR62] Tournier, I. (2022). Learning and adaptation in older adults: An overview of main methods and theories. *Learning, Culture and Social Interaction,**37*, Article 100466. 10.1016/j.lcsi.2020.100466

[CR63] Unal, E., & Cakir, H. (2021). The effect of technology-supported collaborative problem solving method on students’ achievement and engagement. *Education and Information Technologies*, *26*(4), 4127–4150. 10.1007/s10639-021-10463-w

[CR64] van Dijk, J. A. G. M. (2020). *The digital divide*. Polity Press.

[CR65] Varier, D., Dumke, E. K., Abrams, L. M., Conklin, S. B., Barnes, J. S., & Hoover, N. R. (2017). Potential of one-to-one technologies in the classroom: Teachers and students weigh in. *Educational Technology Research and Development,**65*, 967–992.

[CR66] Villar, F., Celdrán, M., Pinazo, S., & Triadó, C. (2010). The teacher’s perspective in older education: The experience of teaching in a university for older people in Spain. *Educational Gerontology*, *36*(10–11), 951–967. 10.1080/03601277.2010.485037

[CR76] Vroman, K. G., Arthanat, S., & Lysack, C. (2015). “Who over 65 is online?” Older adults’ dispositions toward information communication technology. *Computers in Human Behavior, 43*, 156–166. 10.1016/j.chb.2014.10.018

[CR72] Vygotsky, L. S. (1978). *Mind in society: The development of higher psychological processes.* In M. Cole, V. John-Steiner, S. Scribner, & E. Souberman (Eds.), Cambridge, MA: Harvard University Press.

[CR67] Wanya, C. S. (2016). Performance and determinants of problem solving among college physics students. *International Journal of Advanced Research in Management and Social Sciences,**5*(6), 830–854.

[CR68] Whittle, S. R., & Bickerdike, S. R. (2015). Online preparation resources help first year students to benefit from practical classes. *Journal of Biological Education,**49*(2), 139–149.

